# Male Courtship Behavior and Weapon Trait as Indicators of Indirect Benefit in the Bean Bug, *Riptortus pedestris*

**DOI:** 10.1371/journal.pone.0083278

**Published:** 2013-12-27

**Authors:** Yû Suzaki, Masako Katsuki, Takahisa Miyatake, Yasukazu Okada

**Affiliations:** 1 Graduate School of Environmental Science, Okayama University, Okayama, Japan; 2 Faculty of Life and Environmental Science, University of Tsukuba, Ibaraki, Japan; 3 Graduate School of Environmental and Life Science, Okayama University, Okayama, Japan; 4 Department of General Systems Studies, University of Tokyo, Tokyo, Japan; National Cancer Institute, United States of America

## Abstract

Females prefer male traits that are associated with direct and/or indirect benefits to themselves. Male–male competition also drives evolution of male traits that represent competitive ability. Because female choice and male–male competition rarely act independently, exploring how these two mechanisms interact is necessary for integrative understanding of the evolution of sexually selected traits. Here, we focused on direct and indirect benefits to females from male attractiveness, courtship, and weapon characters in the armed bug *Riptortus pedestris*. The males use their hind legs to fight other males over territory and perform courtship displays for successful copulation. Females of *R*. *pedestris* receive no direct benefit from mating with attractive males. On the other hand, we found that male attractiveness, courtship rate, and weapon size were significantly heritable and that male attractiveness had positive genetic covariances with both courtship rate and weapon traits. Thus, females obtain indirect benefits from mating with attractive males by producing sons with high courtship success rates and high competitive ability. Moreover, it is evident that courtship rate and hind leg length act as evaluative cues of female choice. Therefore, female mate choice and male–male competition may facilitate each other in *R*. *pedestris*. This is consistent with current basic concepts of sexual selection.

## Introduction

Female mate choice is one of two major mechanisms of sexual selection and is a fundamental and critical force leading to the evolution of behaviors and morphologies [1]–[7]. Female mate choice occurs when females are selective in their mating decisions and favor attractive males as prospective mates [2], [3], [7]–[9]. Traits such as ornaments for attracting mates are the result of female choice and have been suggested to act as cues to females of direct and indirect benefits [3]. Obvious examples of direct benefit include access to territory, nuptial gifts, and conjoined efforts in parental care for offspring [10], [11]. Females can increase their longevity and/or fecundity from the direct benefits [3], [12].

Indirect benefits also augment female fitness in two general ways (reviewed in [13]–[17]). Firstly, attractive males produce sons that inherit their father's attractiveness and therefore also have increased mating success, and hence female fitness is indirectly increased via their sons' mating success (Fisherian mating advantages: [7], [8], [17], [18]). Secondly, attractive males are themselves of high genetic quality and hence viability, and the offspring sired by these males also inherit the “good genes” [13], [16], [17], [19]. In this case, female fitness is indirectly increased through the production of offspring with high viability (reviewed in [13], [19]).

Male-male competition is classified as another mechanism of sexual selection and occurs when males compete for access to prospective mates [1]–[4]. This process favors exclusion of rival males by highly competitive males, resulting in evolutionary exaggerations of weapon characters, such as the antlers of deer and mandibles of beetles [3], [20], [21]. Indeed, many empirical studies have demonstrated that fighting success is positively impacted by larger weapon size (reviewed in [22], [23]). Although male-male competition and female mate choice have been the focus of a considerable amount of research separately, both mechanisms of sexual selection rarely act independently (reviewed in [24]).

Current concepts of sexual selection basically assume that competitively superior males confer direct and/or indirect fitness benefits on females [25]–[27]. For example, highly competitive males may protect the female during and after mating or provide access to superior resources [28]. Also there is an indirect genetic benefit if weapon traits are heritable [29]. Under such situations, it is expected that selective forces through male-male competition and female mate choice act together in a reinforcing manner.

However, several recent studies suggest that male competition and female choice sometimes work in opposing or unrelated directions [30]–[34], and thus the two mechanisms do not always act in a reinforcing manner. Furthermore, each mechanism occasionally selects for different traits, and as a result, multiple sexual traits play different roles in reproductive behavior even in the same individual, for example, one trait is used for courtship behavior and another for male fighting [33], [35]–[40]. So far, relatively few studies have investigated whether competitively superior males are preferred by females and confer fitness benefits on females [6]. Thus, investigations of interaction between male-male competition and female choice are necessary for integrative understanding of sexual selection (i.e., total sexual selection).

Here, we focused on whether higher successful males in male-male competition are preferred by females, and how mating with preferred attractive males confers fitness benefits on females. Males of *Riptortus pedestris* usually fight for territories using their enlarged hind legs [41], and males with larger weapons tend to win the contest [42], [43]. In contrast, there is little information about male attractiveness and female preference in this species. Numata et al. [44] suggested that male attractiveness is associated with manners of courtship behavior (e.g., foreleg and/or body rubbing). Similar findings are reported for several insects [32], [45]–[48]. Thus, courtship may also act as an evaluative cue of female choice in *R. pedestris*.

In the present study, we investigated whether male attractiveness (i.e., copulation latency), courtship behavior, and weapon size are heritable and how these characters are genetically correlated using full-sib/half-sib analysis methods. Moreover, we examined whether mating with attractive males affects female fitness (lifetime reproductive success and longevity).

## Materials and Methods

### Insect culture

The stock population was cultured from approximately 50 individuals collected in Fukuyama City, Hiroshima, Japan, in late autumn 2006 [49]. Insects were reared on soybean seeds, red clover (*Trifolium pratense*) seeds, and water containing ascorbic acid (0.05%) [50]. Food and water were replaced once every two weeks. The stock was maintained at 1500–2000 nymphs per generation and kept in plastic cups (diameter 95 mm, height 40 mm) with a standing density of between 10 and 20 individuals per cup. After eclosion, each adult was housed in a separate petri dish (90 mm diameter, 15 mm depth). Thus, adults did not interact with conspecifics until the following experiments. For a more detailed description of the stock culture, see Okada et al. [42]. We performed all rearing and experiments in a chamber maintained at 25°C, 60% relative humidity and with a photoperiod cycle of 16∶8 h light∶dark.

### Methods for measurement of copulation latency, courtship rate, and morphology

A virgin male and a virgin female were chosen randomly and placed in a plastic cup (78 mm diameter, 43 mm height) lined with a paper filter (78 mm diameter). Subsequently, the pair was continuously observed until copulation ended using a digital video camera (Victor GZ-MG880). If we did not observe a successful copulation for two hours, the pair was excluded from the analysis. All observations took place between 1500–2300.

In the laboratory, Numata et al. [44] observed a highly stereotypical sequence of courtship behaviors as follows. A male first mounts a female's back and subsequently taps her antenna with his foreleg while shaking his body. When a female accepts the male's mating attempt, she opens her ovipositor valves and the male inserts his genitalia. After genital connection, the male turns around and the pair takes the end-to-end position [44].

In this study, we used courtship rate (number of such courtship bouts per second) as an indicator of courtship quality (e.g., [32], [51]). Furthermore, copulation always occurs after the courtship behavior in *R*. *pedestris*. In these insects, because females generally mate sooner with more attractive males, copulation latency (the time from initiation of courtship to commencement of copulation) is often used as an indicator of male attractiveness (also see [34], [47], [48], [52]), and we thus measured copulation latency as male attractiveness. We noted the repeatability of the courtship rate and copulation latency of *R*. *pedestris* which measured along with the above mentioned methods (courtship rate, *r* = 0.469, *P* = 0.0003, *N* = 52; copulation latency, *r* = 0.648, *P*<0.0001, *N* = 52, Pearson's correlation coefficient).

After mating, each individual was immediately removed from the plastic cup to prevent additional matings. The hind femur length (an estimator of hind leg length) of each male (±0.01 mm) was measured by using a dissecting microscope monitoring system (VM-60; Olympus, Tokyo, Japan). Each specimen was positioned so that its longitudinal and dorsoventral axes were perpendicular to the visual axes of the microscope eyepiece. The length was measured as a straight-line distance (see [43] for landmarks). Each character was measured twice, and the average value was used in the analyses.

### Sib analyses of male attractiveness, courtship rate, and hind leg

To examine genetic variance and covariance, we conducted the following experiment using a full sib/half sib experimental design. Males (sires) (N = 30) were randomly assigned to at least two virgin females (dams) (N = 78) and were allowed mate in the manner described above. After mating, each dam was immediately removed from the plastic container and placed in a petri dish (90 mm diameter, 20 mm deep) containing an excess of food and water. Each female was maintained for four weeks to obtain her offspring. All offspring were reared to adulthood under laboratory conditions identical to the parental generation. A total of 232 sons (mean per dam  = 2.97) were assessed for copulation latency, courtship rate, and morphology in the same manner as described above. Females used for this measurement were chosen randomly from the stock culture.

### Relationships between female preference and direct benefit

To examine whether female fitness is affected by male attractiveness, female longevity and lifetime reproductive success (LRS) were measured. In the beginning, the copulation latency, courtship rate, and male hind leg length of 61 pairs were measured using the above described methods. After mating, each female was placed in a petri dish (90 mm diameter, 20 mm high) containing an excess of food and water and 1 cm^3^ cotton wool as an oviposition site. Egg number and female survival were assessed weekly. The body size of females was measured after death using prothorax width as a proxy [42].

### Statistical analysis

We used a nested model (sire + dam [sire]) for an unbalanced design to estimate the heritability ± SE (*h* ^2^) of each trait [53]. We estimated the genetic correlations ± SE of each trait using the nested model after Falconer and Mackay [54] and Lynch and Walsh [53]. Z scores were used to test whether *h* ^2^ and genetic correlations were significantly different from zero [55], [56].

Because our data showed homogeneity of variances (*F* test: *F*_42_ = 1.184, *P* = 0.586) and normal distribution (Lilliefors test: LRS, *P* = 0.20; longevity, *P* = 0.163), female LRS and longevity were analyzed using a multivariate analysis of variance (MANOVA), with copulation latency and female body size as independent variables. Furthermore, to investigate the effects of courtship rate and male hind leg length on female fitness, we also analyzed female LRS and longevity using MANOVA with courtship rate, male weapon size, and female body size as independent variables. We used backward elimination to remove non-significant interaction terms from the full model [57]. All statistical analyses were carried out using JMP 9.0.2 (SAS Institute).

## Results

**Figure 1:**
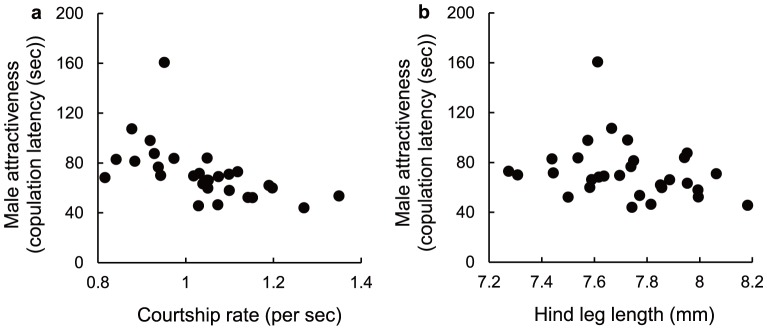
Correlation between male attractiveness and courtship rate and between male attractiveness and hind leg length. The y-axis is the male attractiveness (copulation latency). The x-axis is the courtship rate (a) and hind leg length (b), respectively. Each circle shows family means for each sire. Increases on the y-axis represent decreased attractiveness.

All traits measured were significantly heritable and showed moderate sire heritabilities (Table 1). Copulation latency had significant negative genetic correlations with courtship rate and hind leg size (Fig. 1; Table 1). On the other hand, genetic correlations for other pairs were not significant (Table 1). Because our results included the one highest point (Fig. 1), we reanalyzed the data by excluding the highest point and the statistical significance did not differ from before exclusion (Table 1).

**Table 1:** Heritabilities (*h* ^2^) and genetic correlations ± SE in male attractiveness and traits.

	Latency	Courtship rate	Hind leg length
Latency	**0.822±0.175**		
	**(0.552±0.116)**		
Courtship rate	**−0.539±0.105**	**0.681±0.143**	
	**(−0.693±0.078)**	**(0.721±0.153)**	
Hind leg length	**−0.490±0.114**	0.005±0.148	**0.742±0.156**
	**(−0.561±0.103)**	(0.005±0.150)	**(0.740±0.157)**

Heritabilities (*h* ^2^) are given on the diagonal and additive genetic correlations below the diagonal. Values in parentheses are estimates re-calculated by excluding the highest point of latency. Estimates significantly different from zero (*P*<0.05) are shown in bold.

**Figure 2:**
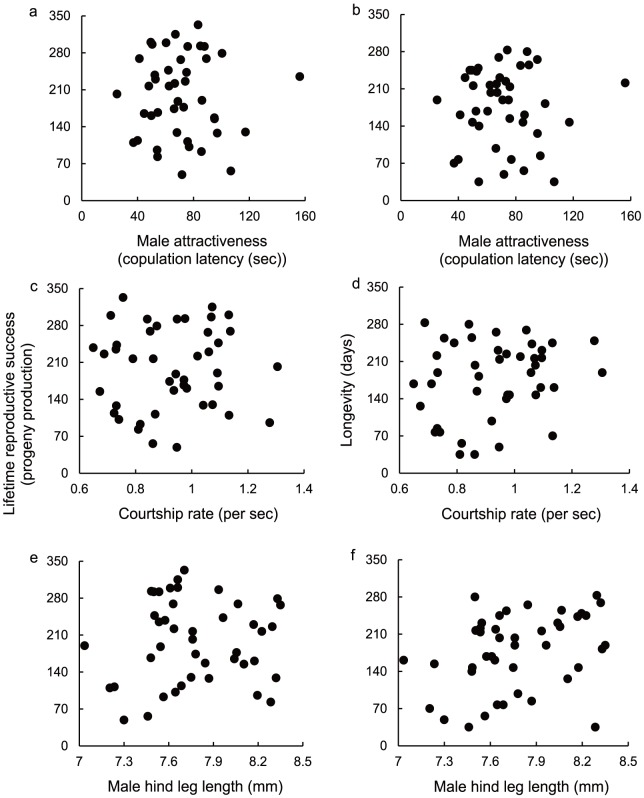
Male attractiveness, courtship rate and hind leg length did not indicate direct benefits on females. Male attractiveness (copulation latency), courtship rate, and male hind leg length were not associated with female lifetime reproductive success and longevity. The y-axis is the female lifetime reproductive success (a, c, e) and longevity (b, d, f), respectively. The x-axis is the Male attractiveness (a, b), courtship rate (c, d), and male hind leg length (e, f), respectively. In male attractiveness, increases on the x-axis represent decreased attractiveness.

Of 61 pairs, successful copulations were observed in 43 pairs. In MANOVA including a copulation latency variable, the reduced model showed that both copulation latency and female body size had non-significant effects (copulation latency, *F*_1, 40_ = 0.0106, *P* = 0.92; female body size, *F*_1, 40_ = 0.0012, *P* = 0.97; Fig. 2a, b). In MANOVA including variables of courtship rate and male weapon size, the reduced model also showed that neither courtship rate, male weapon size nor female body size had significant effect on female LRS and longevity (courtship rate, *F*_1, 39_ = 0.6914, *P* = 0.41; Fig. 2c, d; male weapon size, *F*_1, 39_ = 1.9926, *P* = 0.17; female body size, *F*_1, 39_ = 0.0063, *P* = 0.94; Fig. 2e, f). For each MANOVA, we note that there were non-significant interactions among pairs.

## Discussion

The results showed that males preferred by females tended to have a higher frequency of courtship display and larger hind leg (Table 1, Fig. 1). Furthermore, male attractiveness and male sexual characters (i.e., courtship display and weapon size) had significant heritability (Table 1, Fig. 1). However, mating with more attractive males increased neither LRS nor longevity of females (Fig. 2).

In many insects, females tend to have increased lifetime fitness because of access to male-derived resources (e.g., sperm, nuptial gifts) that elevate fertility/fecundity (reviewed in [58], [59]). However, males are unlikely to supply nutritional advantages to their mates during mating in *R*. *pedestris* (Suzaki, unpublished data). Females can also gain direct benefit from resources or territories possessed by males [3], [12]. For example, in red-collared widowbirds, *Euplectes ardens*, a dominant male can monopolize the best territories required by females for breeding [35]. However, males of *R*. *pedestris* fight for soy bean plants as territories, although females do not stay in the territory after copulation (Suzaki, personal observation). Thus, our results suggest that mating with attractive males brings no direct benefit to *R*. *pedestris* females.

Generally, indirect benefits have a much lower effect than direct benefits [60]–[62]. Nevertheless, when direct benefits are absent, indirect benefits should be required to maintain preference [3], [63]. In this case, females can obtain indirect benefits via their son's mating success or increasing viability of their offspring (e.g., [47], [52], [64], [65]). Because no direct fitness benefit was found in *R*. *pedestris*, indirect benefits may maintain female preference even if this effect is small (also see [66]). Indeed, there was a significant heritability in male attractiveness (copulation latency) in this bug and hence the females can produce attractive sons when mated with attractive males. This is consistent with the Fisherian process and contributes to maintenance of female preference [7], [8], [18].

We found a positive genetic association between male attractiveness and courtship rate (Table 1, Fig. 1). This suggests that the courtship rate acts as an evaluative cue of female choice. These courtship behaviors can also play a critical role in mating success in other insect species (e.g., [30], [51], [52], [67]–[69]). By contrast, male courtship displays sometimes negatively impact female fitness as male sexual harassment [70], [71]. However, mating with males delivering high courtship benefits did not affect female fitness in *R*. *pedestris* (Fig. 2c, d), and thus the female is unlikely to be under such situations. On the other hand, because of a significant heritable variation of the courtship rate, females can sire sons delivering high courtship rates when they mated with attractive males. Production of sons with high courtship rates will contribute indirect benefits to the female.

A similar trend is found in male hind leg size (Table 1, Fig. 1). Weapon size is positively associated with competitive ability for mates [42], [43]. In this species, males establish their territories on soybean plants [41] and call over conspecific individuals irrespective of sexes by aggregation pheromone [72]. When other males encroach, the territory holders fight against intruder, whereas when females are attracted, they court to females and attempt to mate [41]. Because it takes sucking soy bean to secrete aggregation pheromone [72], establishment and defense of territory are critical for their mating success. Therefore, the females can also sire sons highly successful in not only female mate choice but also male-male competition when they mate with the attractive males who have larger weapons (also see [28], [29]).

There was no significant genetic correlation between courtship rate and hind leg length (Table 1). This suggests that the characters are not functionally linked and act independently as evaluative cues of male quality or conditions: the courtship behavior and hind leg length reflect abilities to secure more rapid successful copulation and to compete for mates, respectively. Recent studies suggest that females use multiple cues to choose their mates [33], [36], [38], [39], [73], [74]. This is because use of multiple cues can reduce assessment errors and cost of choice (reviewed in [38]). Females of *R*. *pedestris* may also use multiple cues for mate choice in order to acquire higher quality males who provide larger indirect benefit to their mates.

Our results showed that male-male competition and female mate choice facilitate each other, and this is consistent with current concepts of sexual selection [25]–[29]. However, several studies found that males that are successful in competition are not always successful in female choice [6], [31], [33], [37], [39], [75] because more competitive males frequently impair female fitness [24], [75]. In this situation, selective forces of male competition and female choice may not be reinforcing [76]. Again, we note that the female of this species is unlikely to be harmed by a male because they do not stay in the male's territory after copulation. Furthermore, mating with highly competitive males did not affect female fitness (Fig. 2e, f). Therefore, the female of *R*. *pedestris* is unlikely to be under such situations.

Finally, we observed relatively high heritabilities of both sexually selected traits (Table 1). Theoretical predictions expected little genetic variance in sexually selected traits because directional selection is assumed to drive beneficial alleles to fixation and therefore a particular genotype should become predominant [54], [77], [78]. It is proposed several hypotheses which maintain genetic variance in these fitness-related traits (reviewed in [78]). For example, genetic trade-offs between fitness-related traits should act to preserve the genetic variance in these traits [53], [79]. Otherwise, condition dependence is predicted to contribute genetic variance of these characters [78], [80]. Indeed, sexually selected traits can be costly for males [81]–[83], resulting in genetic trade-off between sexually selected traits and other traits (e.g., [84]–[87]). Moreover, many studies demonstrated that sexually selected traits are condition dependent (reviewed in [87], [88]). In future, it is necessary to investigate genetic trade-offs and condition dependence of traits of male *R*. *pedestris*, to order to reveal how the genetic variance of sexually selected traits is maintained.

In summary, the courtship rate and hind legs act as an evaluative cue of female choice in *R*. *pedestris*. A female may obtain indirect benefit from mating with attractive males, but there were no direct benefits for female *R*. *pedestris* at least as far as we investigated. The indirect benefits in part consist of production of sons with high courtship rates and high competitive ability. The females may use multiple cues to choose their mate in order to maximize the indirect benefits they obtain.
